# Supplementary material for: Large-scale genetic analysis reveals mammalian mtDNA heteroplasmy dynamics and variance increase through lifetimes and generations
Source: Nat Commun. 2018 Jun 27;9:2488. doi: 10.1038/s41467-018-04797-2 (PMC6021422; doi:10.1038/s41467-018-04797-2)
Supplement: Supplementary file 1 — Supplementary Information [file 41467_2018_4797_MOESM1_ESM.pdf]

Large-scale genetic analysis reveals mammalian mtDNA heteroplasmy dynamics and variance increase through lifetimes and generations

Burgstaller *et al*.

Supplementary Information

Supplementary Note 1

Baseline linear model for variance with time.

When considering heteroplasmy variance alone, we use a simple linear model $V'(h) = \varphi t + \theta + \varepsilon$. The constant θ accounts for transient effects including the rapid increase of variance in early development due to the mtDNA bottleneck[1]. ε is a normally-distributed noise term, the standard deviation of which we infer with the other parameters in the model. This linear increase with rate φ is predicted by theory[2]. We use bootstrapping with the percentile method[3] to derive confidence intervals for these parameters.

Model fit statistics

In the section "Time-dependent mtDNA heteroplasmy segregation", the maximum likelihood and 95% c.i.s of the rates of change of heteroplasmy (in day^{-1} units) are: LE oocyte -4.3×10^{-4} (-9.9×10^{-4} to 9.8×10^{-5}; n.s.); LE pup 3.7×10^{-4} (-9.9×10^{-4} to 1.7×10^{-3}; n.s.); HB oocyte -1.6×10^{-4} (-5.9×10^{-4} to 2.7×10^{-4}; n.s.); HB pup -1.3×10^{-3} (-2.3×10^{-3} to -2.9×10^{-4}; $p = 0.025$). Most observed changes are thus limited. The likelihood ratio test in that section compares a null model where the same linear trend describes both mother and pup behaviour (H_0) and an alternative model where mother and pup follow different linear trends with time (H_1). In both these models we allow variance to increase with time.

In the section "Heteroplasmy variance increases linearly over lifetimes", maximum likelihood inferred values and 95% confidence intervals for the weighted-fit rate of change are, in day^{-1} units, (HB oocytes) 1.7×10^{-4} (1.0×10^{-4} to 2.6×10^{-4}, $p < 10^{-3}$); (HB pups) 2.5×10^{-5} (-7.9×10^{-5} to 2.1×10^{-4}, n.s.); (LE oocytes) 2.3×10^{-4} (1.4×10^{-4} to 3.3×10^{-4}, $p < 10^{-3}$); (LE pups) 1.2×10^{-4} (1.7×10^{-5} to 2.2×10^{-4}, $p = 0.029$).

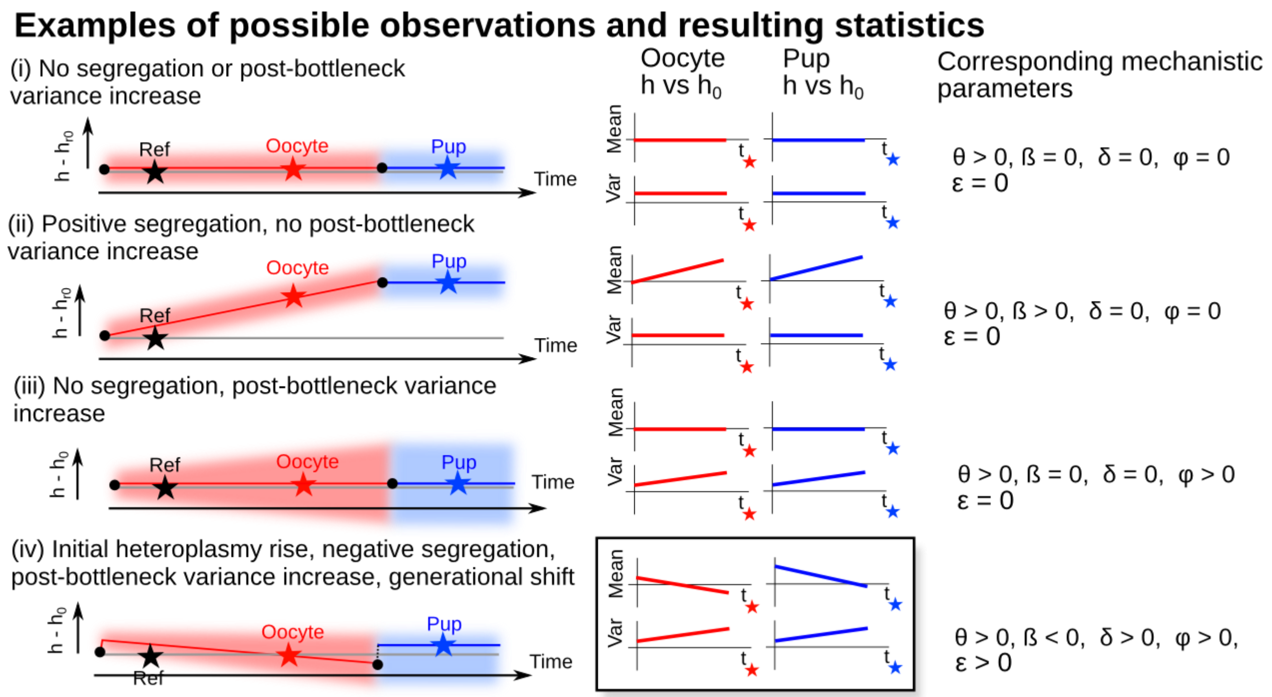

Supplementary Figure 1. **Possible mechanisms governing mtDNA populations.** This figure illustrates how different combinations of mechanistic parameters within our stochastic model would give rise to different observed patterns of heteroplasmy mean and variance over time in our observed dataset. Case (iv), highlighted, describes one situation for which we find statistical support in the full dataset.

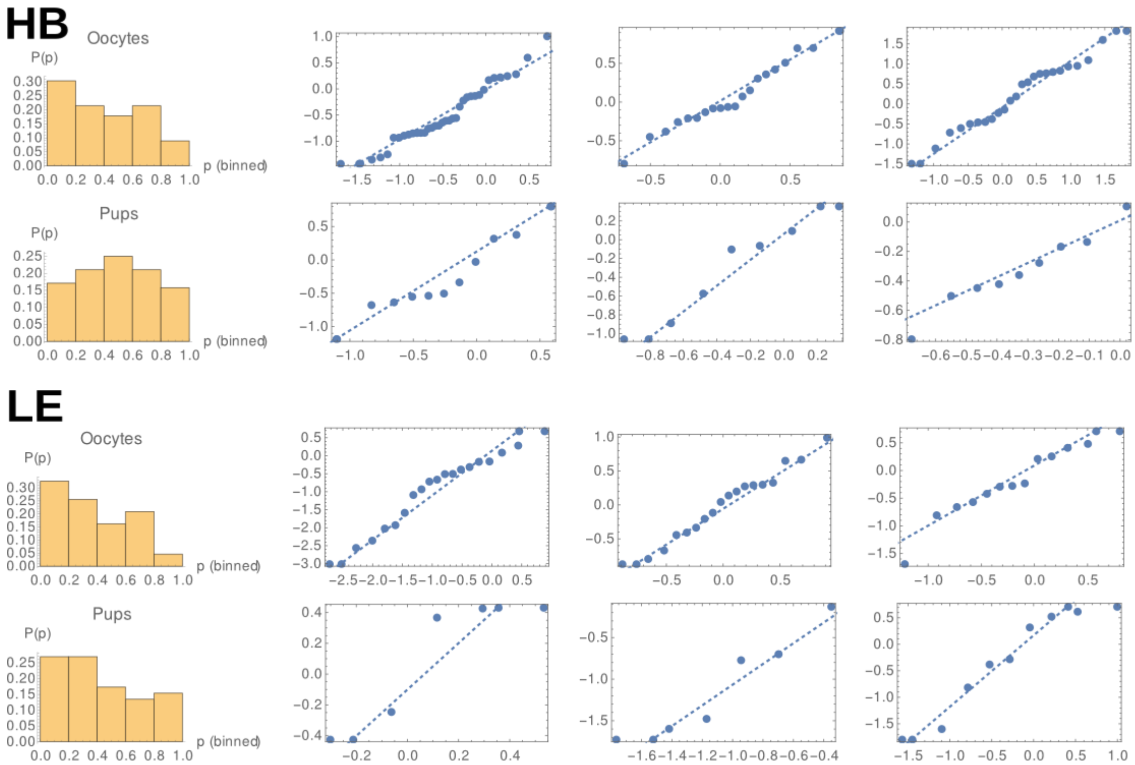

Supplementary Figure 2. **Normality of transformed heteroplasmy distributions.** Histograms give distribution of p-values from Kolmogorov-Smirnov normality tests of individual transformed oocyte and pup distributions. Qq plots show a representative selection of three transformed oocyte and three transformed pup distributions from each set of haplotype samples.

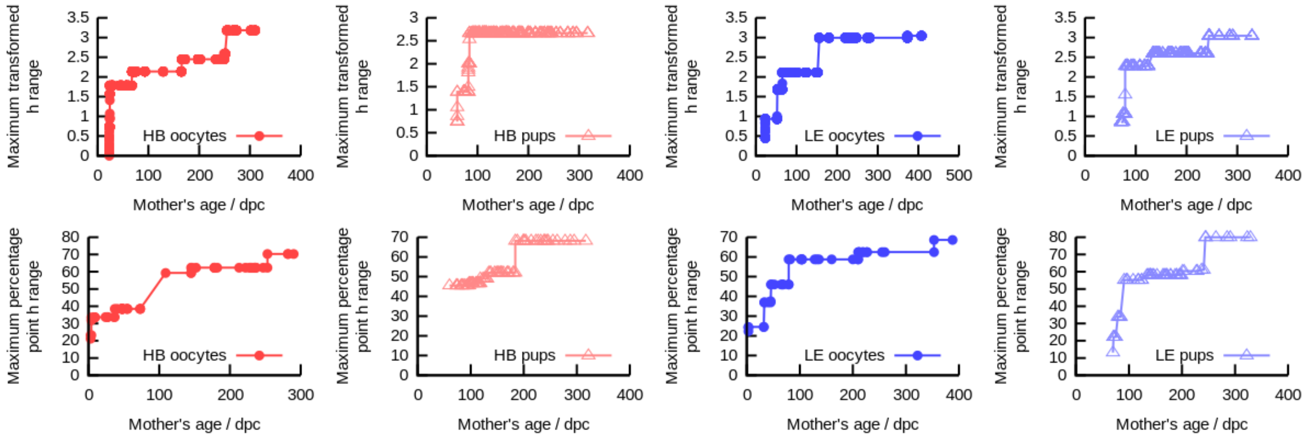

Supplementary Figure 3. **Heteroplasmy range with age.** (top) Maximum transformed heteroplasmy shift with age in oocytes and pups from different models with age (age of female for oocytes, age of mother at birth for pups). (bottom) Maximum percentage point range (maximum minus minimum) in heteroplasmy. This plot is illustrative; percentage point range should be interpreted cautiously, as it depends on the absolute heteroplasmy values involved (hence our use of transformed values throughout).

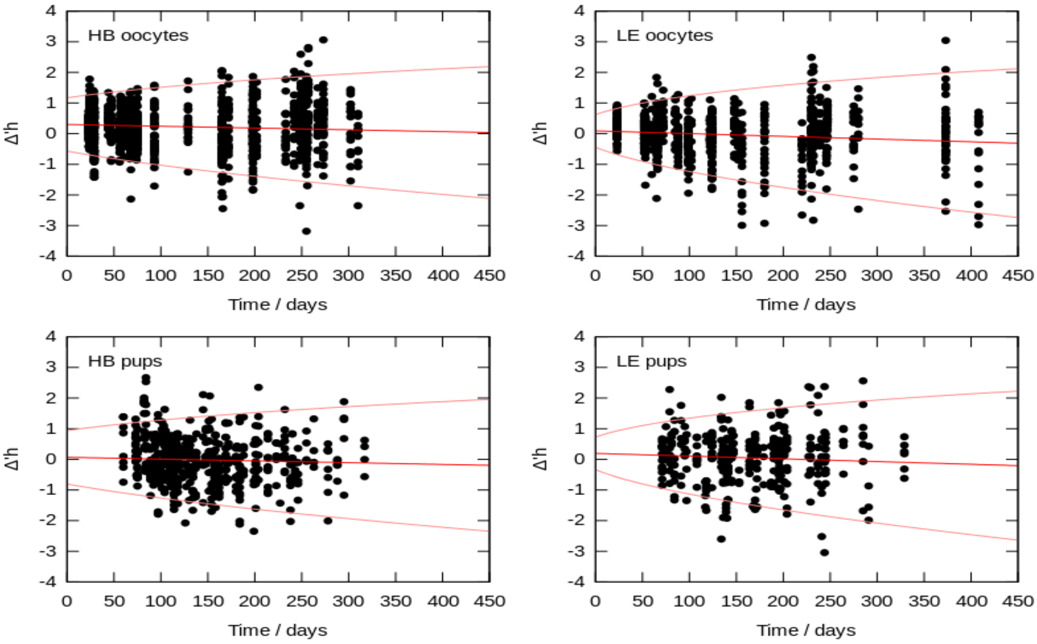

Supplementary Figure 4. **Confidence intervals on predicted heteroplasmy shifts.** Datapoints show transformed heteroplasmy changes for each haplotype pairing and for oocytes and pups. Traces show modelled mean and 95% c.i.s with time (corresponding to a linear increase in variance over time), from parameter values inferred in the main text.

Supplementary References

1. Johnston IG*, et al.* Stochastic modelling, Bayesian inference, and new in vivo measurements elucidate the debated mtDNA bottleneck mechanism. *Elife* **4**, e07464 (2015).

2. Johnston IG, Jones NS. Evolution of cell-to-cell variability in stochastic, controlled, heteroplasmic mtDNA populations. *Am J Hum Genet* **99**, 1150-1162 (2016).

3. Wasserman L. *All of statistics: a concise course in statistical inference*. Springer Science & Business Media (2013).
