# Supplementary material for: Large-scale genetic analysis reveals mammalian mtDNA heteroplasmy dynamics and variance increase through lifetimes and generations
Source: Nat Commun. 2018 Jun 27;9:2488. doi: 10.1038/s41467-018-04797-2 (PMC6021422; doi:10.1038/s41467-018-04797-2)
Supplement: Supplementary file 3 — Description of Additional Supplementary Files [file 41467_2018_4797_MOESM3_ESM.pdf]

Description of Additional Supplementary Files

File Name: Supplementary Data 1
Description: Raw heteroplasmy measurements from HB and LE mouse models, labelled by model, type (oocyte or pup), age, and sex of mice.
